# Diagnostic values of chest pain history, ECG, troponin and clinical gestalt in patients with chest pain and potential acute coronary syndrome assessed in the emergency department

**DOI:** 10.1186/s40064-015-0992-9

**Published:** 2015-05-07

**Authors:** Arash Mokhtari, Eric Dryver, Martin Söderholm, Ulf Ekelund

**Affiliations:** Department of Internal Medicine, Skåne University Hospital at Lund, Lund, Sweden; Department of Emergency Medicine, Skåne University Hospital at Lund, Lund, Sweden; Department of Clinical Sciences at Lund, Section of Emergency Medicine, Lund University, Lund, Sweden

**Keywords:** Acute coronary syndrome, Chest pain, ECG, Diagnosis, Gestalt, Probability

## Abstract

In the assessment of chest pain patients with suspected acute coronary syndrome (ACS) in the emergency department (ED), physicians rely on global diagnostic impressions (‘gestalt’). The aim of this study was to determine the diagnostic value of the ED physician’s overall assessment of ACS likelihood, and the values of the main diagnostic modalities underlying this assessment, namely the chest pain history, the ECG and the initial troponin result. 1,151 consecutive ED chest pain patients were prospectively included. The ED physician’s interpretation of the chest pain history, the ECG, and the global likelihood of ACS were recorded on special forms. The discharge diagnoses were retrieved from the medical records. A chart review was carried out to determine whether patients with a non-ACS diagnosis at the index visit had ACS or suffered cardiac death within 30 days. The gestalt was better than its components both at ruling in (“*Obvious ACS*”, LR 29) and at ruling out (“*No Suspicion of ACS*”, LR 0.01) ACS. In the “*Strong suspicion of ACS*” group, 60% of the patients did not have ACS. A positive TnT (LR 24.9) and an ischemic ECG (LR 8.3) were strong predictors of ACS and seemed superior to pain history for ruling in ACS. In patients with a normal TnT and non-ischemic ECG, chest pain history typical of AMI was not a significant predictor of AMI (LR 1.9) while pain history typical of unstable angina (UA) was a moderate predictor of UA (LR 4.7). Clinical gestalt was better than its components both at ruling in and at ruling out ACS, but overestimated the likelihood of ACS when cases were assessed as strong suspicion of ACS. Among the components of the gestalt, TnT and ECG were superior to the chest pain history for ruling in ACS, while pain history was superior for ruling out ACS.

**Electronic supplementary material:**

The online version of this article (doi:10.1186/s40064-015-0992-9) contains supplementary material, which is available to authorized users.

## Background

Non-traumatic chest pain is a common presenting complaint among patients seeking care in the Emergency Department (ED). The management of these patients often hinges upon the perceived likelihood that an acute coronary syndrome (ACS) accounts for the patient’s chest pain. A substantial proportion of patients with chest pain are admitted for inpatient care in order to rule-out ACS, and many undergo stress testing, of which only a small proportion are abnormal and lead to a change in management (Penumetsa et al. 2012). These admissions and investigations in patients without ACS cause a substantial health care burden (Goodacre et al. 2005). At the same time, 2-4% of patients with ACS are erroneously discharged from the ED (Lee et al. 1987; Pope et al. 2000). These patients have a higher mortality than patients with ACS who are admitted, further emphasizing the need for an improved assessment of the likelihood of ACS in the emergency department (Lee et al. 1987; Pope et al. 2000).

The main tools used to determine the likelihood of ACS in the ED are the chest pain history, the ECG and blood markers of myocardial injury such as troponins. The predictive values for ACS of these diagnostic methods have been extensively analyzed (Chun and McGee 2004; Panju et al. 1998; Swap and Nagurney 2005; Lee et al. 1985), but the studies have mostly focused on single items (e.g. radiation to the arms) and on diagnosing acute myocardial infarction (AMI) and not ACS. Single items are insufficient predictors of ACS for the purpose of ED decision-making (Swap and Nagurney 2005), and a number of clinical prediction rules combining items have therefore been developed (Christenson et al. 2006; Fesmire et al. 2012; Hess et al. 2012; Six et al. 2008). However, the value of these prediction rules in ED routine care has not yet been established (Hess et al. 2008; Manini et al. 2009; Steurer et al. 2010). Most clinicians instead rely on a global, subjective patient assessment known as ‘gestalt.’ Studies have demonstrated that the clinical gestalt for pulmonary embolism performs at least as well as clinical prediction rules (Chunilal et al. 2003; Runyon et al. 2005; Penaloza et al. 2013). Also, the clinical gestalt for acute cholecystitis has a high predictive accuracy even in the absence of individual findings with high predictive power (Trowbridge et al. 2003). The diagnostic accuracy of the clinical gestalt for ACS is unclear.

Knowledge of the diagnostic accuracy of the gestalt for ACS may help ED clinicians to make better decisions when managing patients with chest pain. The aim of this study was to determine the diagnostic value of the ED physician’s overall clinical assessment of ACS likelihood, and the values of the main diagnostic modalities underlying this assessment, namely the chest pain history, the ECG and the initial troponin result.

## Methods

### Study site

The Skåne University Hospital at Lund is a tertiary care, 700 bed institution that serves as the primary hospital for some 300,000 inhabitants. Percutaneous coronary intervention (PCI) and coronary artery bypass surgery (CABG) are available 24 hours a day. Roughly 65,000 patients are assessed every year in the ED, of which about 5,500 present with non-traumatic chest pain. There is no dedicated chest pain observation unit. Patients with ST-elevation myocardial infarction (STEMI) who are identified via ambulance ECGs as a rule bypass the ED and are taken directly to the angiography suite.

### Patient population

All patients aged over 18 years who presented with non-traumatic chest pain to the Lund ED during June 12th - October 8th 2009 were prospectively identified and enrolled in the study. Patients were excluded from the analysis if the history was unreliable due to e.g. alcohol intoxication or dementia, if they were transferred to another hospital, if they refused admission for inpatient evaluation, or if data were missing.

### Routine clinical assessment

All included patients were initially assessed by a triage team that measured vital signs, recorded an ECG and took routine blood tests including a troponin T (TnT). The patients were then assessed by a resident or a specialist in internal and/or emergency medicine. This physician took a history, performed a physical exam, and if necessary, reviewed the case with a senior colleague.

### Data collection

After the patient encounter, the physician or one of the authors (MS) recorded the physician’s assessment of the patient on a specific study form (see Additional file 1). The assessments were all made at the same time.

First, the physician categorized the chest pain history as *typical of AMI, typical of Unstable Angina (UA), nonspecific for ACS*, or *not suspicious of ACS*. The form specified that central, pressure-type pain lasting over 15 minutes with or without radiation to the arm or shoulder is considered typical for AMI. Specifications for the other categories were not provided. Next, the physician noted the presence or absence of the following ECG changes: a) ST-elevation or depression ≥ 1 mm in at least two anatomically contiguous leads; b) left bundle branch block (LBBB); c) pathological Q-waves in at least two anatomically contiguous leads; d) T-wave inversions in at least two anatomically contiguous leads. In the present study, a non-ischemic ECG was defined as an ECG lacking all of the findings above.

Last, physicians recorded their composite assessment of the likelihood of ACS based on the chest pain history, ECG and the first TnT value, which in principle was always available at the time of assessment. In order to limit heterogeneity of the assessments, physicians had to choose among four ACS likelihood levels, with suggested definitions provided on the data form: *Obvious ACS*, typical symptoms and ST-elevation or LBBB not previously observed; *Strong suspicion of ACS,* a) typical symptoms or b) ST-T changes or LBBB not previously observed, or c) acute heart failure or hypotension regardless of ECG or d) ventricular tachycardia/fibrillation or AV-block III; *Low suspicion of ACS,* unclear symptoms and history, non-ischemic ECG; and *No suspicion of ACS*, a) no suspicion of ischemic heart disease, or b) stable angina pectoris. The physicians were free to disregard these definitions, but the definitions where non-controversial and reflected common clinical reasoning at the hospital during the study.

The troponin assay used in this study was Elecsys troponin T, which has a 99th percentile cutoff of 0.01 μg/L, and with 0.03 μg/L reported as the lowest concentration with a coefficient of variation ≤ 10%. The first TnT test result was retrieved from the electronic patient records, with values ≥ 0.05 μg/L being considered indicative of ACS.

### Outcome measures

Patients admitted after the ED assessment were cared for by ward physicians blinded to the data form. The discharge diagnosis (ICD 10) was obtained from the discharge summary, which was written by the ward physician and reviewed for quality and accuracy by a specialist in internal medicine and/or cardiology. For patients discharged from the ED, the discharge diagnosis (ICD 10) was retrieved from the ED patient record written by the responsible ED physician. Patients were considered to have ACS if they received the diagnosis in the Skåne University Hospital’s patient records, or suffered a cardiac death during the index visit or within 30 days of ED presentation. Those who received a non-ACS diagnosis at the index visit and were diagnosed with ACS within 30-days were categorized as missed ACS.

### Statistics and ethics

All measures of diagnostic performance (sensitivity, specificity, likelihood ratios) were calculated with ACS as the outcome measure, except chest pain history assessed as “*typical of AMI*” and *“typical of UA”,* where AMI and UA were used as the respective outcome measures. When a cell of a 2 x 2 table was 0, 0.5 was added to all cells before calculating likelihood ratios (LR) (Chun and McGee 2004). Analyses were made using IBM SPSS Statistics v19 and Microsoft Excel 2007. All included patients gave informed consent in writing, and the study was approved by the regional ethics committee in Lund (DNR 2009/630).

## Results

### Patient characteristics

**Figure 1:**
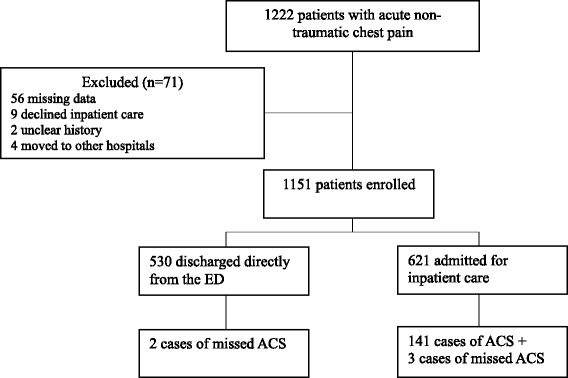
Flow diagram of enrolled and excluded patients.

As shown in Figure 1, 1,222 patients were included in the study. Seventy-one patients were excluded based on predefined criteria, leaving 1,151 patients in the final analysis. The baseline characteristics of these patients are listed in Table 1. The mean age was 61 years, 29% had a history of coronary artery disease (CAD), and 15% had diabetes. Fifty-four per cent of the patients were admitted for inpatient care but only 23% of these had ACS. In the entire study population, 13% had a final diagnosis of ACS (97 AMI, 49 UA) during the index visit or within 30 days. In the remaining patients, the most common causes of chest pain were unspecified chest pain, musculoskeletal pain, and stable angina. One case of AMI and four cases of UA were missed according to our criteria, implying a 3.4% miss rate.

**Table 1:** **Baseline patient characteristics and physician assessments**

	**All patients** ***n = 1151 (%)***	***% with ACS n = 146 (97 MI, 49 UA)***
**Admitted to hospital**	621 (54)	23.0
**Age (years)**		
<40	177 (15.4)	0
40-65	474 (41.2)	10.3
>65	500 (43.4)	19.4
>80	176 (15.3)	21.0
**Gender**		
Male	646 (56.1)	16.9
**Cardiovascular history**		
Known PAD	26 (2.3)	23.1
Diabetes	168 (14.6)	25.0
Previous stroke	103 (8.9)	22.3
Known CAD	338 (29.4)	22.5
Known heart failure	116 (10.1)	12.1
**Chest pain history**		
Typical of ACS	327 (28.4)	38.5
Typical of AMI	147 (12.8)	36.7
Suspicious of UA	180 (15.6)	40.0
Nonspecific	409 (35.5)	4.6
Not suspicious of ACS	415 (36.1)	0.2
**ECG**		
ST elevation	23 (2.0)	69.6
ST depression	46 (4.0)	63.0
T-wave inversion	35 (3.0)	34.3
Q-wave	3 (0.3)	33.3
LBBB	8 (0.7)	37.5
**Troponin T, initial**		
Elevated ≥ 0.05 μg/L	78 (6.8)	78.2
**Overall clinical gestalt**		
Obvious ACS	21 (1.8)	81.0
Strong suspicion	250 (21.7)	41.2
Low suspicion	439 (38.1)	5.9
No suspicion	441 (38.3)	0

PAD, peripheral artery disease; CAD, Coronary artery disease.

ACS, Acute coronary syndrome; AMI, Acute myocardial infarction; UA, Unstable angina.

LBBB, Left bundle branch block.

### Diagnostic performance of cardiac risk factors

As can be seen in Table 2, age < 40 years almost excluded ACS (LR 0.02) while higher age increased the ACS probability only slightly. A previous history of CAD, peripheral artery disease (PAD), stroke and diabetes did not alter post-test probability of ACS.

**Table 2:** **Diagnostic performance of cardiac risk factors in percent (95% CI) for ACS within 30 days**

	**Sensitivity**	**Specificity**	**LR+**	**LR-**
**Age (years)**				
<40	0.3 (0.0-3.2)	82 (80–85)	0.02 (0.00-0.31)	1.2 (1.2- 1.2)
40-65	34 (27–42)	58 (55–61)	0.8 (0.6-1.0)	1.1 (1.0-1.3)
>65	66 (58–73)	60 (57–63)	1.6 (1.4-1.9)	0.6 (0.5-0.7)
>80	25 (19–33)	86 (84–88)	1.8 (1.3-2.5)	0.9 (0.8-1.0)
**Risk factors**				
Known PAD	4 (2–9)	98 (97–99)	2.0 (0.8-5.1)	1.0 (0.9-1.0)
Diabetes	29 (23–37)	88 (85–89)	2.4 (1.8-3.2)	0.8 (0.7- 0.9)
Previous stroke	16 (11–23)	92 (90–94)	2.1 (1.4-3.2)	0.9 (0.8-1.0)
Known CAD	51 (43–59)	74 (71–76)	2.0 (1.6-2.4)	0.7 (0.6-0.8)
Known heart failure	9 (5–15)	90 (88–91)	0.9 (0.5-1.5)	1.0 (1.0-1.1)
Male gender	75 (67–81)	47 (44–50)	1.4 (1.3-1.6)	0.5 (0.4- 0.7)

LR, Likelihood ratio; PAD, peripheral artery disease; CAD, Coronary artery disease.

### Diagnostic performances of likelihood assessments

The diagnostic performances of the assessments are described in Tables 3 and 4. Chest pain history judged by the physician as typical of AMI increased the probability of AMI (LR 4.9, Table 3). However, when excluding patients with ischemic ECG changes and elevated initial troponins (Table 4), chest pain history deemed typical of AMI had only a small impact on the likelihood of AMI (LR 1.6). Symptoms suspicious of UA also increased the probability of a final diagnosis of UA (LR 5.6), but in contrast retained its predictive ability even when there were no ischemic ECG changes and the initial TnT was normal (LR 4.7). Meanwhile, symptoms assessed as not suspicious of ACS almost excluded the diagnosis (LR 0.02). The presence of any ischemic ECG changes or an elevated TnT both increased the probability of ACS markedly (LR 7.6 and 24.9 respectively), while their absence only had a minimal effect (LR 0.6 and 0.7). The clinician’s overall assessments of obvious or strong suspicion of ACS significantly raised the probability (LR 29 and 4.8) of ACS, whereas no suspicion of ACS practically ruled out the diagnosis (LR 0.01).

**Table 3:** **Diagnostic performance of physician assessments in percent (95% CI) for ACS within 30 days**

	**Sensitivity**	**Specificity**	**LR+**	**LR-**
***Chest pain history***				
Typical of ACS	86 (80–91)	80 (77–82)	4.3 (3.8-5.0)	0.2 (0.1-0.3)
Typical of AMI±	47 (38–57)	90 (88–92)	4.9 (3.7-6.5)	0.6 (0.5-0.7)
Typical of UA≠	73 (60–84)	87 (85–89)	5.6 (4.5-7.1)	0.3 (0.2-0.5)
Nonspecific for ACS	13 (8–19)	61 (58–64)	0.3 (0.2-0.5)	1.4 (1.3-1.5)
Not suspicious of ACS	1 (0–4)	59 (56–62)	0.02 (0.00-0.12)	1.7 (1.6-1.8)
***ECG***				
ST-elevation	11 (7–17)	99 (99–100)	15.7 (6.6-37.6)	0.9 (0.8-0.9)
ST-depression	20 (14–27)	98 (97–99)	11.7 (6.6-20.8)	0.8 (0.8-0.9)
T-wave inversion	8 (5–14)	98 (97–98)	3.6 (1.8-7.1)	0.9 (0.9-1.0)
Non-Ischemic*	59 (51–67)	5 (4–6)	0.6 (0.5-0.7)	7.6 (5.5-10.6)
***TnT***				
Positive initial TnT	42 (34–50)	98 (97–99)	(15.0-41.5)	0.7 (0.5- 0.7)
***Overall suspicion of ACS***				
Obvious ACS	12 (7–18)	100 (99–100)	29 (10–86)	0.9 (0.8-0.9)
Strong suspicion	71 (63–77)	85 (83–87)	4.8 (4.0-5.8)	0.4 (0.3-0.4)
Low suspicion	18 (12–25)	59 (56–62)	0.4 (0.3-0.6)	1.4 (1.3-1.5)
No suspicion	0.3 (0.0-3,2)	56 (53–59)	0.01 (0.00-0.12)	1.8 (1.7-1.9)

ACS, Acute coronary syndrome; LR, likelihood ratio; TnT, Troponin T; UA, Unstable angina.

± Calculated with AMI as outcome measure.

≠ Calculated with UA as outcome measure.

*Defined as absence of ST-elevation, ST-depression, T-wave inversion, q-waves and LBBB.

**Table 4:** Diagnostic performances of chest pain history in percent (95% CI) in patients with non-ischemic ECG* and normal initial troponin T

	**Sensitivity**	**Specificity**	**LR+**	**LR-**
***Chest pain history***				
Typical of ACS	32 (25–39)	83 (81–86)	1.9 (1.4-2.5)	0.8 (0.7-0.9)
Typical of AMI±	11 (6–19)	93 (91–94)	1.6 (0.9-3.0)	1.0 (0.9-1.0)
Typical of UA≠	45 (32–59)	90 (89–92)	4.7 (3.3-6.7)	0.6 (0.5-0.8)
Nonspecific for ACS	6 (3–11)	64 (61–67)	0.2 (0.1-0.3)	1.5 (1.4-1.6)
Not suspicious of ACS	0.3 (0–3)	60 (57–63)	0.01 (0.00-0.13)	1.7 (1.6-1.8)

LR, likelihood ratio; ACS, Acute coronary syndrome; AMI, Acute myocardial infarction; UA, Unstable angina pectoris.

*Normal ECG defined as the absence of ST-elevation, ST-depression, T-wave inversion, q-waves and LBBB.

± Calculated with AMI as outcome measure.

≠ Calculated with UA as outcome measure.

## Discussion

In this prospective study of patients with non-traumatic chest pain, we analyzed the diagnostic values of the overall clinical assessment of ACS likelihood, and the values of the main diagnostic modalities underlying this assessment, namely the chest pain history, the ECG and the initial troponin result. Our main findings were three: *First,* age < 40 years, chest pain history and overall gestalt not suspicious of ACS all practically ruled out ACS. *Second,* a positive initial TnT and an ischemic ECG were strong predictors of ACS and seemed superior to pain history for ruling in ACS. *Third,* in patients with a normal initial TnT and non-ischemic ECG, chest pain history typical of AMI was not a significant predictor of AMI while chest pain history typical of UA was a moderate predictor of UA.

The present study shows, not surprisingly, that the overall clinical gestalt was better than its components both at ruling in (“*Obvious ACS*”, LR 29) and at ruling out (“*No Suspicion of ACS*”, LR 0.01) ACS. None of the 441 patients with a “*No Suspicion of ACS*” gestalt had ACS within 30 days. In accordance with the results by Kline et al. 2014 (Kline and Stubblefield), a “*No Suspicion*” gestalt thus seems to rule out ACS in the ED, and to obviate the need for admission, serial troponins, and stress-testing for the exclusion of ACS. Miller et al. found that 2.8% of ED patients assessed as “noncardiac chest pain” had adverse cardiac events within 30 days (Miller et al. 2004). However, in that study TnT was not measured in all patients and the gestalt impression was recorded before biomarkers were drawn. About half of their patients with adverse cardiac events turned out to have elevated troponins meaning they would not have been classified in the “*No Suspicion of ACS*” group in our study. A “*No Suspicion of ACS*” gestalt and a non-suspicious chest pain history both almost excluded ACS, and one might speculate that the ED physicians based their no suspicion gestalt primarily on the chest pain history. Our results thus suggest that a non-suspicious pain history may in many cases be enough to rule out ACS in the ED, at least if the pretest probability is low. Only 0.2% of the patients with a non-suspicious pain history had ACS within 30 days in our population. Further, the study confirms previous findings that age < 40 years argues strongly against ACS (Collin et al. 2011; Marsan et al. 2005), while older age has limited predictive value (Chun and McGee 2004).

We have found four previous publications with data on the diagnostic or prognostic value of the overall clinical gestalt in patients with possible ACS. However, two of the studies were from before the modern biomarker era and focused on AMI only (Karlson et al. 1991; Tierney et al. 1986), one was much smaller than the present study and included patients without chest pain and/or TnT tests (Ekelund et al. 2002), and one was primarily prognostic and included only low risk patients (Chandra et al. 2009). In the present study, the gestalt had largest predictive ability when cases were assessed as *“Obvious ACS”* or *“No suspicion of ACS”*. In the strong suspicion group, it appeared that the physicians’ gestalt overestimated the likelihood of ACS, since 60% of these patients did not have ACS. The value of a high grade of suspicion of ACS may thus be less than generally believed. In this context, Kline et al. 2014 reported that emergency physicians tend to overestimate the likelihood of ACS also in low risk patients (Kline and Stubblefield). In the low suspicion gestalt group 6% of the patients had ACS, which indicates that these patients should in general undergo further evaluation.

In accordance with previous findings, a negative initial TnT and a non-ischemic ECG did not reliably rule-out ACS (Chun and McGee 2004; Ebell et al. 2000; Fesmire et al. 1989). On the other hand, TnT and ECG seemed superior to chest pain history for ruling in ACS. Our findings thus support the practice of admitting all chest pain patients with ischemic ECGs and/or elevated TnT for additional evaluation. In patients with a non-ischemic ECG and a negative initial TnT, a pain history typical of AMI was poorly predictive of AMI (LR 1.6). In contrast, pain typical of UA was still a moderate predictor of UA (LR 4.7). The results thus indicate that patients with a pain history typical of UA should undergo further evaluation, regardless of the ECG and TnT results, which is probably true even if highly sensitive troponins are used (Borna et al. 2014).

### Limitations of the study

This study was performed at only one university hospital and the results are not necessarily generalizable to other hospitals. However, the prevalence of ACS among chest pain patients was 12.7%, which is comparable to that in other studies of unselected ED chest pain patients (Han et al. 2007; Scheuermeyer et al. 2012).

The discharge diagnoses were those used in routine clinical care. Since we aimed to study diagnostic value in routine care, we did not assess the diagnoses for accuracy, and we have no data on what proportion were based on objective testing, e.g. stress tests or coronary angiography. However, at our institution which is the academic cardiac center for the entire region, most patients are evaluated with stress testing and virtually all patients with ACS undergo coronary angiography. All discharge diagnoses were reviewed for quality and accuracy by the attending specialist physician (most often cardiologist, in a few cases internal medicine specialist). In addition, the patients were followed for 30 days after the ED visit. The discharge diagnoses reflected real life practice, and we believe that very few were inaccurate.

In our review of the patient records at 30 days to determine whether an ACS diagnosis was missed or if the patient died, we may have missed a small number of patients presenting to other hospitals. However, such misclassifications were probably few and unlikely to significantly affect the results of this study.

In the analysis, patients with or without ongoing chest pain were not separated. We have no data as to whether they were evaluated or treated differently.

Suggested definitions of the different levels of ACS suspicion were present on the study forms, and although they left considerable room for judgment, other (or no) definitions may have led to somewhat different results. The definition of typical symptoms of MI might have been suboptimal as it is somewhat non-specific, but it is a definition commonly used in guidelines (Amsterdam et al. 2010). Although the physicians were instructed to disregard ECG and TnT when evaluating the symptoms, we cannot exclude that ECG and TnT results influenced the symptom assessment in some cases.

As TnT was used in the gestalt assessment as well as in deciding the final diagnosis, incorporation bias could have been present. This was however probably limited by the fact that the emergency physicians only had access to the initial TnT, whereas the discharge diagnoses were most often based on repeated TnT analyses to assess for significant rise or fall.

Finally we did not have data regarding physician level of experience. However, at least in the assessment of pulmonary embolism, differences in the diagnostic accuracy of the gestalt depending on experience are small (Kabrhel et al. 2005).

### Suggestions for further studies

Many of our results have broad confidence intervals suggesting that a larger study with a similar aim would be preferable in order to confirm the findings.

Several clinical decision support tools and risk prediction scores for patients with suspected ACS have been published, e.g. the HEART score (Six et al. 2008). For any such tool or score to be clinically useful, they have to be at least as good as the gestalt. We suggest that future studies compare new decision support tools and scores with the physician’s gestalt assessment. Interestingly, it has been shown that the gestalt performs better than the Wells score in the assessment of the probability of pulmonary embolism (Penaloza et al. 2013).

## Conclusion

Not surprisingly, gestalt was better than its components both at ruling in (LR 29) and at ruling out (LR 0.01) ACS. The gestalt seemed to overestimate the likelihood of ACS when cases were assessed as strong suspicion of ACS. Among the components of the gestalt, the initial TnT and ECG were superior to the chest pain history for ruling in ACS, while pain history was superior for ruling out ACS. In patients with a non-ischemic ECG and a normal TnT, a chest pain history typical of AMI was not a significant predictor of AMI, but a pain typical of UA was still a moderately good predictor of UA.

## Additional file

Additional file 1:
**Study form.**

## Abbreviations

ACS: Acute coronary syndrome
AMI: Acute myocardial infarction
CABG: Coronary artery bypass grafting
CAD: Coronary artery disease
ECG: Electrocardiogram
ED: Emergency department
LBBB: Left bundle branch block
LR: Likelihood ratio
PAD: Peripheral artery disease
PCI: Percutaneous coronary intervention
STEMI: St-elevation myocardial infarction
TnT: Troponin t
UA: Unstable angina
