# Supplementary material for: Diagnostic values of chest pain history, ECG, troponin and clinical gestalt in patients with chest pain and potential acute coronary syndrome assessed in the emergency department
Source: Springerplus. 2015 May 7;4:219. doi: 10.1186/s40064-015-0992-9 (PMC4431985; doi:10.1186/s40064-015-0992-9)
Supplement: Additional file 1: — Study form. [file 40064_2015_992_MOESM1_ESM.doc]

**Chest pain**

The patient’s part

We are conducting a study to evaluate our current way of assessing chest pain patients. We would be grateful if you would answer the following questions. If anything is unclear do not hesitate to ask us for help. Thank you for your cooperation!

**Date. . . . . . . . . . . . . . . Time. . . . . . . . . .**

***Prior illnesses and medications:***

*Yes No*

| Patient-ID |
| --- |

*Have you ever had a coronary bypass operation or coronary catheter intervention?*

***Do you have:***

*Poor blood circulation in the legs/ claudication?*

*Angina?*

*Diabetes?*

*Heart failure?*

*Atrial fibrillation?*

*Yes No*

*Have you had a stroke?*

*Do you take warfarin?*

*Do you receive treatment for elevated cholesterol?*

*If* ***Yes****, which drugs are you taking? (write below)*

*…………………………………………………………….*

The physician’s part

*Please answer these questions!*

1. ***Assessment of symptoms***

***(Only symptoms!)***

*Symptoms typical of MI (Pressing, central chest pain > 15 min, ± radiation to arms/shoulders.)*

*Symptoms typical of unstable angina.*

*Symptoms nonspecific for ACS.*

*Symptoms not suspicious for ACS.*

1. ***Clinical exam***

*Yes No*

*Bilateral basal rales*

*Bilateral rales > halfway up the lung fields*

*Pitting edema*

*Elevated jugular venous pressure*

*Pulmonary edema*

*Cardiogenic shock*

1. ***ECG (findings not known to be old)***

*Yes No*

*ST elevation? (>1 mm in > 2 adjacent leads)*

*ST depression? (>1 mm in > 2 adjacent leads)*

*Left bundle branch block?*

*Q-waves? (pathological in > 2 adjacent leads)*

*T-neg?( in > 2 adjacent leads)*

*Atrial fibrillation?*

*Assessment of the ECG:………………………………………*

*..................................................................................*

1. ***Overall probability of ACS (Choose only one!)***

***Obvious ACS:*** *typical symptoms and ST-elevation or LBBB not previously observed.*

***Strong suspicion of ACS:***

1. *Typical symptoms or,*
2. *ST-T changes or LBBB not previously observed or,*
3. *Acute heart failure or hypotension regardless of ECG or,*
4. *Ventricular tachycardia/fibrillation or AV-block III*

***Low suspicion of ACS****: unclear symptoms and history, non-ischemic ECG.*

***No suspicion of ACS***:

1. *No suspicion of ischemic heart disease.*
2. *Stable angina pectoris.*
